# Brentuximab Vedotin for Treating Relapsed or Refractory CD30-Positive Cutaneous T-Cell Lymphoma: An Evidence Review Group Perspective of a NICE Single Technology Appraisal

**DOI:** 10.1007/s41669-020-00203-0

**Published:** 2020-03-23

**Authors:** Angela Stainthorpe, Nigel Fleeman, Rachel Houten, Marty Chaplin, Angela Boland, Sophie Beale, Yenal Dundar, Joanne McEntee, Isabel Syndikus

**Affiliations:** 1 Liverpool Reviews and Implementation Group, University of Liverpool, Whelan Building, Brownlow Hill, Liverpool, L69 3GB UK; 2 North West Medicines Information Centre, Liverpool, L69 3GF UK; 3 Faculty of Health and Life Sciences, University of Liverpool, Thompson Yates Building, Liverpool, L69 3GB UK; 4 The Clatterbridge Cancer Centre NHS Foundation Trust, Bebington, Wirral CH63 4JY UK

## Abstract

As part of the single technology appraisal process, the National Institute for Health and Care Excellence invited Takeda UK Ltd to submit clinical- and cost-effectiveness evidence for brentuximab vedotin (BV) for treating relapsed or refractory CD30-positive (CD30+) cutaneous T-cell lymphoma (CTCL). The Liverpool Reviews and Implementation Group at the University of Liverpool was commissioned to act as the evidence review group (ERG). This article summarises the ERG’s review of the company’s submission for BV and the appraisal committee (AC) decision. The principal clinical evidence was derived from a subgroup of patients with advanced-stage CD30+ mycosis fungoides (MF) or primary cutaneous anaplastic large-cell lymphoma (pcALCL) in the phase III ALCANZA randomised controlled trial (RCT). This trial compared BV versus physician’s choice (PC) of methotrexate or bexarotene. Evidence from three observational studies was also presented, which included patients with other CTCL subtypes. The ERG’s main concerns with the clinical evidence were the lack of RCT evidence for CTCL subtypes other than MF or pcALCL, lack of robust overall survival data (data were immature and confounded by subsequent treatment and treatment crossover on disease progression) and lack of conclusive results from analyses of health-related quality-of-life data. The ERG noted that many areas of uncertainty in the cost-effectiveness analysis were related to the clinical data, arising from the rarity of the condition and its subtypes and the complexity of the treatment pathway. The ERG highlighted that the inclusion of allogeneic stem-cell transplant (alloSCT) as an option in the treatment pathway was based on weak evidence and generated more uncertainty in a disease area that, because of its rarity and diversity, was already highly uncertain. The ERG also lacked confidence in the company’s modelling of the post-progression pathway and was concerned that it may not produce reliable results. Results from the company’s base-case comparison (including a simple discount patient access scheme [PAS] for BV) showed that treatment with BV dominated PC. The ERG’s revisions and scenario analyses highlighted the high level of uncertainty around the company base-case cost-effectiveness results, ranging from BV dominating PC to an incremental cost-effectiveness ratio per quality-adjusted life-year gained of £494,981. The AC concluded that it was appropriate to include alloSCT in the treatment pathway even though data were limited. The AC recommended BV as an option for treating CD30+ CTCL after at least one systemic therapy in adults if they have MF, stage IIB or higher pcALCL or Sézary syndrome and if the company provides BV according to the commercial arrangement (i.e. simple discount PAS).

## Key Points for Decision Makers

**Table:** 

Randomised controlled trial (RCT) evidence for the effectiveness of brentuximab vedotin was only available for two subtypes of cutaneous T-cell lymphoma (CTCL): mycosis fungoides and primary cutaneous anaplastic large-cell lymphoma.
Overall survival (OS) data from the RCT were immature and confounded by subsequent anticancer therapy and treatment crossover, meaning that the reliability of results from analysis of OS data was highly uncertain
Inclusion of allogenic stem-cell transplant (alloSCT) as an option in the cost-effectiveness analysis was based on weak evidence and generated further uncertainty in a disease area that, because of its rarity and diversity in presentation, was already highly uncertain.
The structure of the post-progression health state and alloSCT relapse health state in the company’s economic model prevented investigation of alternative OS assumptions and led to implausible outcomes for patients following certain treatment pathways. There was considerable uncertainty surrounding resource use, costs and time spent in the post-progression health state and alloSCT relapse health state.

## Introduction

The National Institute for Health and Care Excellence (NICE) is an independent organisation responsible for providing guidance to the national health service (NHS) in England and Wales on a range of clinical and public health issues and appraising new health technologies. The NICE single technology appraisal (STA) process is designed for appraisal of a single health technology for a single indication, where most of the relevant evidence lies with one company or sponsor and typically covers new technologies shortly after UK market authorisation is granted [1]. Within the STA process, the company provides a written submission (including a decision-analytic model) that summarises the company’s estimate of the clinical and cost effectiveness of the technology. Consultees, clinical specialists and patient representatives also provide additional information during the appraisal process.

Using a specification developed by NICE (the final scope), the NICE appraisal committee (AC) considers the company’s submission [2], the evidence review group (ERG) report and testimonies from experts and stakeholders to determine whether the technology represents a clinically and cost-effective use of NHS resources. All stakeholders and the public have an opportunity to comment on the preliminary guidance issued by NICE in the form of an appraisal consultation document (ACD), after which the AC meets again to produce the final guidance (final appraisal determination [FAD]). The final guidance, if positive, constitutes a legal obligation for NHS providers in England and Wales to provide the recommended technology within its licensed indication [1].

This article presents a summary of the ERG report produced by the Liverpool Reviews and Implementation Group at the University of Liverpool for the STA of brentuximab vedotin (BV) for treating relapsed or refractory cluster of differentiation 30-positive (CD30+) cutaneous T-cell lymphoma (CTCL). Takeda UK Ltd was the sponsoring company for this STA. Full details of all relevant appraisal documents (including the appraisal scope, ERG report, company and consultee submissions, NICE guidance and comments on each of these) can be found on the NICE website [2].

## The Decision Problem

### Underlying Health Problem

Primary cutaneous lymphomas are a class of non-Hodgkin’s lymphoma that starts in the skin [3]. The majority of primary cutaneous lymphomas are CTCLs (75–80% in the Western world [3]). The incidence of CTCL is low (reported to be 0.7 per 100,000 from 2009 to 2013 in the UK [4] and 1.02 per 100,000 from 2005 to 2009 in the USA [5]), and CTCL meets EU criteria for designation as an orphan disease (prevalence of fewer than five people per 10,000 [6]). Men are diagnosed with CTCL at around 1.6 times the rate of women [5], although this varies by disease subtype [7]. Median age at diagnosis is between 55 and 60 years [8].

The focus of this appraisal was on patients with the following subtypes of CTCL: mycosis fungoides (MF; the most common CTCL variant, accounting for around 60% of CTCLs), Sézary syndrome (SS; approximately 2% of CTCLs [3]) and primary cutaneous CD30+ lymphoproliferative disorders (LPDs), comprising primary cutaneous anaplastic large-cell lymphoma (pcALCL) and lymphomatoid papulosis (LyP) (8% and 12% of CTCLs, respectively [3]).

Severity of MF can be categorised as early (stage IA–IIA) or advanced (stages IIB–IVB), based on tumour-node-metastasis-blood (TNMB) designations [9]. MF usually presents at an early stage and is often slow growing [10]. Progression to advanced-stage MF occurs in around 25% of patients [4]. Advanced-stage MF is characterised by skin tumours, erythroderma and nodal or visceral involvement [11, 12]. SS is a leukaemic and aggressive form of CTCL closely related to MF. SS presents only in advanced-stage disease with extreme pruritus, erythroderma, lymphadenopathy and circulating Sézary cells [13]. Patients with pcALCL generally present with rapidly growing and ulcerating large tumours or thick plaques (which are localised solitary or grouped) [14, 15]. Extracutaneous spread (i.e. metastasis) is reported to occur in 13% of patients with pcALCL [14, 15]. Patients with LyP typically present with recurrent nodules and papules at distant sites, which become necrotic before resolving to form an atrophic scar [13, 15].

While CTCL is nearly always incurable, prognosis for patients with early-stage MF, pcALCL or LyP is generally good. However, overall survival (OS) varies by CTCL subtype and disease severity. Five-year OS rates have been reported as 88% for patients with all stages of MF, 24% for patients with SS [16], ≥ 83% for patients with pcALCL [17] and ≥ 90% for patients with LyP [13, 14, 18]. For patients with MF, life expectancy falls dramatically as MF progresses. Five-year OS rates have been reported to vary from 97% for patients with stage IA MF and 18% for those with stage IVB MF/SS [4]. Similarly, 5-year OS rates have been reported to vary from 91% for patients with localised pcALCL and 50% for those with generalised pcALCL [14].

### Current Management Strategies

Patients with CTCL are managed primarily according to subtype of CTCL and stage of disease according to published guidelines [10, 15, 19–21].

Early-stage CTCL tends to be managed expectantly (i.e. ‘watch and wait’) or with skin-directed therapies [4, 10, 15, 19–21]. Therapies can include application of topical treatments, localised radiotherapy, psoralen plus ultraviolet A light therapy, narrow-band ultraviolet B or a combination of these treatments.

Systemic treatment options for advanced-stage MF or SS include biological agents, chemotherapy and allogenic stem-cell transplantation (alloSCT). AlloSCT is a form of immunotherapy aimed at producing durable complete remission [4, 22].

The company in this appraisal followed the National Comprehensive Cancer Network’s naming scheme by classifying therapies for advanced CTCL as category A or B treatments [22]. Category A therapies are generally used before category B therapies. Table 1 shows the types of category A and B therapies available in the UK.

**Table 1:** Category A and B therapies

Category A therapies	Category B therapies
Interferon-α Methotrexate Bexarotene Extracorporeal photopheresis	Single-agent chemotherapy regimens, most notably gemcitabine or pegylated liposomal doxorubicin (not available at all centres) Multi-agent chemotherapy regimens, most notably cyclophosphamide, doxorubicin, vincristine and prednisone (CHOP) Total skin electron beam therapy

Source: Company submission, Figure 14, published guidelines [10, 15, 19–21] and review [39]

There is a paucity of comparative efficacy data and a lack of consensus on a preferred systemic therapy [21, 23]. Therefore, initial choice of treatment is generally made by the clinician on an individual patient basis according to their needs and the expertise of the centre. AlloSCT tends to be an option for patients with advanced MF or SS with good performance status who are responding to treatment [4, 22].

### Intervention

The treatment considered in this appraisal was BV. BV is an antibody–drug conjugate that delivers an antineoplastic agent, resulting in apoptotic cell death selectively in CD30-expressing tumour cells. The CD30-targeted mechanism of action means that BV can overcome chemo-resistance. BV is indicated for the treatment of adult patients with CD30+ CTCL after at least one prior systemic therapy [24].

## Independent Evidence Review Group (ERG) Report: Initial Submission

### Clinical Evidence

The comparator specified by NICE in the final scope was established clinical management without BV. The company considered that the relevant comparators to BV were physician’s choice (PC), i.e. methotrexate or bexarotene, both of which are category A comparators (a single-agent chemotherapy and retinoid, respectively). The focus of the company submission (CS) was a subgroup of the licensed population, namely patients with advanced-stage CTCL. The company’s rationale for this approach was that patients with advanced-stage CTCL constituted the population most relevant to NHS clinical practice.

The company presented evidence for the clinical effectiveness of BV from the ALCANZA trial [25]. The ALCANZA trial was an international, open-label, randomised, phase III, multicentre trial of BV versus PC of methotrexate or bexarotene in patients with MF or pcALCL and was the only relevant randomised controlled trial (RCT) of BV identified by the company’s literature searches. Evidence from three single-arm observational studies [26–28] (two of which were prospective phase II studies [26, 28]) was also included in the CS. The observational studies included patients with subtypes other than MF or pcALCL, including SS and LyP. The company assessed the feasibility of performing indirect comparisons to obtain (1) estimates of effectiveness of BV versus interferon (IFN)-α, another category A therapy and (2) estimates of effectiveness of BV versus standard of care for patients with SS/LyP (established clinical management without BV). It was not possible to conduct these indirect comparisons because available data were insufficient.

A total of 131 patients were enrolled into the ALCANZA trial between 13 August 2012 and 31 July 2015 and randomly assigned (1:1) centrally to receive BV (*n* = 66) or PC (*n* = 65). Randomisation was stratified by baseline disease diagnosis (MF or pcALCL). BV was administered intravenously at a dose of 1.8 mg/kg once every 3 weeks, for a maximum of 48 weeks. In the PC arm, patients received oral methotrexate 5–50 mg once weekly or oral bexarotene 300 mg/m^2^ once daily. Patients received methotrexate or bexarotene for up to 48 weeks. Patients were defined as having advanced-stage CTCL if they had a diagnosis of MF stage IIB or later or pcALCL. In total, 49 patients treated with BV and 46 patients treated with PC were classified as having advanced-stage CTCL at baseline (*n* = 95; 73% of all patients in the trial).

Data from the ALCANZA trial were presented from two data cuts: 22.9 and 33.9 months’ median follow-up. Table 2 summarises the key efficacy results for the advanced-stage CTCL patient population from after 33.9-month median follow-up. Results showed that BV resulted in increased objective global response lasting at least 4 months (objective response rate [ORR]-4; primary outcome), increased ORR and improved median progression-free survival (PFS) and time to subsequent anticancer therapy. The company highlighted that OS results were highly uncertain as the data were immature (16 events [33%] in the BV arm and 18 events [39%] in the PC arm) and confounded by subsequent anticancer therapy and crossover; 55% of patients in the BV arm and 63% of patients in the PC arm received subsequent treatments, and 46% of patients in the PC arm subsequently received BV.

**Table 2:** Efficacy results for the ALCANZA trial, subgroup of patients with advanced-stage CTCL (33.9-month median follow-up)

Outcome	BV (*n* = 49)	PC (*n* = 46)
**ORR4**		
*n*	29	4
% (95% CI)	59.2 (45.4–72.9)	8.7 (2.4 to 20.8)
% difference (95% CI) *p* value^a^	50.5 (31.6–66.4) *p* < 0.001	
**PFS**		
Median, months (95% CI)	16.5 (15.5–27.5)	3.5 (2.4 to 4.9)
HR (95% CI)	0.30 (0.18–0.50)	
**Response rates**		
ORR		
*n* % (95% CI)	34 69.4 (56.5–82.3)	8 17.4 (6.4 to 28.3)
% difference (95% CI) *p* value^a^	52.0 (35.1–68.9) *p* < 0.001	
Complete response		
*n* % (95% CI)	10 20.4 (9.1–31.7)	1 2.2 (0.1 to 11.5)
	% difference (95% CI) *p* value^a^	18.2 (− 2.0 to 37.6) *p* = 0.005
Partial response		
*n* (%)	24 (49.0)	7 (15.2)
Stable disease		
*n* (%)	8 (16.3)	12 (26.1)
Progressive disease		
*n* (%)	3 (6.1)	16 (34.8)
Not evaluable		
*n* (%)	4 (8.2)	10 (21.7)
**Time to subsequent anticancer therapy**		
Median, months (95% CI)	14.2 (12.2–20.4)	5.5 (3.4–9.5)
HR (95% CI)	0.31 (0.19–0.51)	
**OS**		
Median, months (95% CI)	43.6 (41.0–NA)	41.6 (21.1–NA)

*BV* brentuximab vedotin, *CI* confidence interval, *CMH* Cochran-Mantel–Haenszel, *CTCL* cutaneous T-cell lymphoma, *HR* hazard ratio, *MF* mycosis fungoides, *NA* not available, *ORR* objective response rate, *ORR4* objective global response lasting ≥ 4 months, *OS* overall survival, *PC* physician’s choice, *PFS* progression-free survival

^a^*p* value calculated using a CMH test stratified by baseline disease diagnosis (pcALCL and MF)

In the subgroup of patients with advanced-stage CTCL in the ALCANZA trial, after a median follow-up of 22.9 months, more patients treated with BV reported any-grade treatment-related adverse events (AEs), treatment-related serious AEs and discontinuations due to AEs than did patients treated with PC. In contrast, more grade ≥ 3 treatment-emergent AEs (TEAEs) were experienced by patients in the PC arm than by patients in the BV arm. Peripheral neuropathy was the most common grade ≥ 3 TEAE for patients treated with BV (14%). Grade ≥ 3 TEAEs were uncommon for patients treated with methotrexate, but grade ≥ 3 hypertriglyceridemia was reported by one-quarter of patients with advanced-stage CTCL treated with bexarotene.

Health-related quality life (HRQoL) data for patients with advanced-stage CTCL were presented from analyses of data derived from the Skindex-29 [29] and the three-level EuroQoL 5-Dimensions (EQ-5D-3L) [30] instruments. The company reported that, after a median follow-up of 33.9 months, patients with advanced-stage CTCL treated with BV had greater skin symptom reduction than those treated with PC. The difference was described as being clinically meaningful. Although patients in the BV arm had higher mean EQ-5D-3L index scores at baseline than patients in the PC arm, there were no notable differences in change from baseline in either treatment arm.

### Critique of the Clinical Evidence and Interpretation

The ERG noted that RCT evidence was only available for two subtypes of CTCL: MF and pcALCL. Supportive evidence from observational data presented in the European Public Assessment Report (EPAR) [31] for BV for patients with other subtypes of CTCL was limited. The ERG highlighted that it was difficult to obtain clinical-effectiveness evidence for patients with subtypes of the condition given the rarity of CTCL, particularly subtypes other than MF.

The ERG noted that the Cox proportional hazards (PH) method was used to estimate hazard ratios (HRs) for the outcomes of PFS and time to subsequent anticancer therapy. However, the ERG considered that the PH assumption may be violated for both these outcomes. Since HRs are not an appropriate summary of treatment effect when the PH assumption does not hold, the ERG considered that the reported HRs for PFS and time to subsequent anticancer therapy should be interpreted with caution.

The ERG agreed with the company that OS results from the ALCANZA trial should be interpreted with caution due to confounding by subsequent anticancer therapy and crossover, the small number of patients in the analysis and the small number of events that had occurred. The ERG also agreed with the company that none of the available methods of crossover adjustment were suitable for the ALCANZA trial given the small number of patients and events and the lack of a secondary common baseline at the time data were collected on time-dependent covariates (a prerequisite for the two-stage method of crossover adjustment). The ERG considered it was not possible to obtain a robust estimate of OS for BV versus PC.

Safety data from the ALCANZA trial showed that, for patients with advanced-stage CTCL, BV was not associated with any new or unexpected toxicities, and most reported AEs were grade 1 or 2 in severity. Clinical advice to the ERG was that peripheral neuropathy is the most common and clinically significant AE associated with treatment with BV. The ERG noted that 86% of patients with peripheral neuropathy experienced either an improvement or resolution, but nine (20%) patients with peripheral neuropathy eventually discontinued treatment with BV.

The ERG concurred with the European Medicines Agency [31] that no firm conclusions about the impact of BV on HRQoL could be drawn.

The ERG agreed with the company that it was not possible to conduct an indirect comparison of BV versus IFNα or of BV versus standard of care for patients with SS/LyP. Restricting the category A comparators to methotrexate and bexarotene was not considered by the ERG to be a major limitation of the evidence base since clinical advice was that methotrexate, bexarotene and IFNα are considered to have equal efficacy.

### Cost-Effectiveness Evidence

The company developed a de novo model in Microsoft Excel to compare the cost effectiveness of BV versus PC for patients with advanced-stage CTCL who had been previously treated with at least one systemic therapy. The model structure comprised five mutually exclusive health states: pre-progression, alloSCT, non-alloSCT post-progression, alloSCT relapse and dead. The non-alloSCT post-progression and alloSCT relapse states were both divided into subsequent therapy and end-stage care phases. The company used a Markov structure with a partitioned survival approach to estimate transitions out of the pre-progression and alloSCT states and assigned estimates of mean time spent in the post-progression and alloSCT relapse states (hereafter referred to as post-progression states) to each transition from the pre-progression and alloSCT states. The model time horizon was 45 years with a 1-week cycle length. The model perspective was that of the UK NHS. Outcomes were measured in quality-adjusted life-years (QALYs). Costs and QALYs were discounted at 3.5% annually.

Patient survival and patient utility were estimated based on data from the 33.9-month median follow-up of the ALCANZA trial. Resource use and costs were estimated based on information from the ALCANZA trial, skin systemic anticancer therapy treatment protocols, other published sources and advice from clinical experts. Costs sources included hospital outpatient appointments, home visits, investigations and test costs from NHS reference costs (2016/17) [32], specialist dressing costs from the British National Formulary (2018) [33], the cost of alloSCT from Debals-Gonthier et al. [34] and end-of-life care costs from Round et al. [35]. A Department of Health patient access scheme (PAS) discount was applied to the price of BV. Full list prices were used for bexarotene [36] and methotrexate [37].

The company used Kaplan–Meier (KM) data from the ALCANZA trial to generate two independent Weibull curves to estimate PFS for patients treated with BV and with PC who did not receive alloSCT. Mean PFS in the company base case for patients who did not receive alloSCT was 1.78 years for BV and 0.59 years for PC. The company fitted a single log-logistic curve to OS KM data from the PC arm of the ALCANZA trial to estimate long-term survival for patients who did not receive alloSCT, regardless of initial treatment. Mean survival in the company model for patients who did not receive alloSCT was 6.83 years.

The company base-case analysis included the assumption that some patients who achieved a complete or partial response to treatment with BV or PC would receive alloSCT after 18 weeks of treatment. Post-alloSCT outcomes were estimated by fitting parametric curves to digitised OS and disease-free survival (DFS) data from a real-world dataset (RWD) [38]. Mean PFS in the company base case (including alloSCT in the pathway) was 1.33 years for BV and 0.54 years for PC. Mean life expectancy in the company base case (including alloSCT in the pathway) was 8.43 years for BV and 7.23 years for PC.

**Fig. 1:**
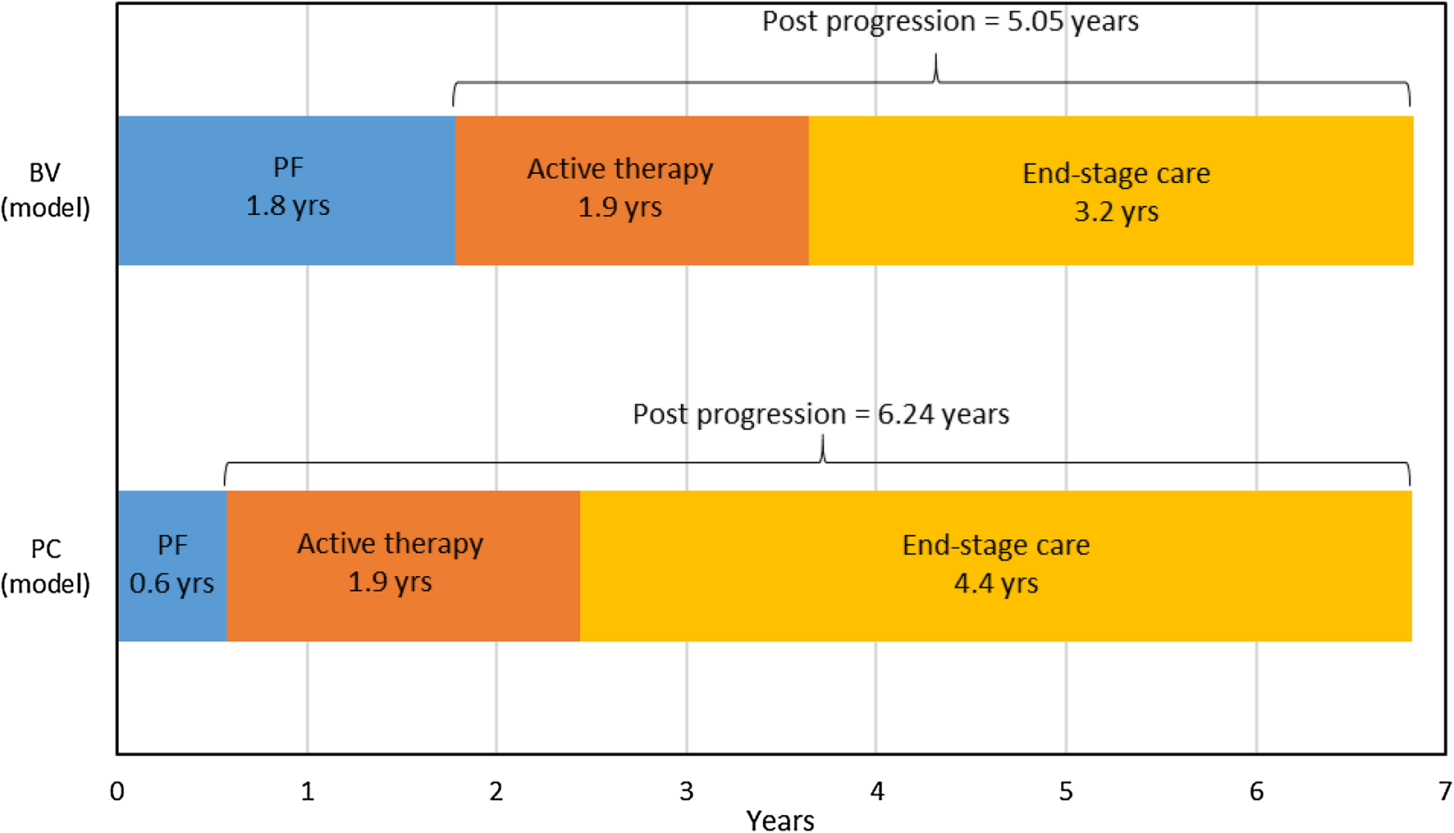
Mean time spent in each health state in the company base-case model without allogeneic stem-cell transplant. *BV* brentuximab vedotin, *PC* physician’s choice, *PF* progression free, *yrs* years

Patients entering the post-progression health states in each cycle were assigned mean costs and QALYs (discounted) based on the estimated mean time spent in each of two post-progression phases: subsequent (active) therapy and end-stage care. Data from the ALCANZA trial were used to estimate mean time spent in the post-progression health states for patients who did not receive alloSCT, and data from the RWD were used to estimate mean time spent in the alloSCT relapse state for patients who received alloSCT. Mean time spent receiving subsequent therapy (1.9 years) was estimated from the literature and assumed to be the same in the post-progression or alloSCT relapse health states. However, if, in the model, mean time in the post-progression health states was shorter than the mean subsequent therapy time parameter, then time receiving subsequent therapy was cut short. Time spent receiving end-stage care varied according to the difference between time spent receiving subsequent therapy and total time spent in the post-progression health states (Fig. 1). Time spent receiving end-stage care could be zero if time spent receiving subsequent treatment in the model was equal to or less than time spent in the post-progression health states.

Complete time on treatment (ToT) data were available from both arms of the ALCANZA trial, and the company adjusted these data to fit within the weekly-cycle structure of the model.

Utility values were estimated using a longitudinal mixed-effects regression model to adjust the EQ-5D-3L data collected during the trial to take into account progression status and Skindex-29 total score. Utility values used in the pre-progression health state differed by primary treatment, whereas, in the progressed disease health state, the same utility value was used irrespective of primary treatment. The utility values in the alloSCT health states and in the post-progression health states were obtained from published sources (Table 3).

**Table 3:** Utility values in the company base case and ERG revised models

State	Utility value: mean		Source
	Company base case	ERG revised base case	
PFS–BV	0.68	0.69	ALCANZA trial
PFS–PC	0.64	0.69	ALCANZA trial
SCT (0–14 days)	0.42	No change	van Agthoven et al. [40]
SCT (14 days–3 months)	0.60	No change	van Agthoven et al. [40]
SCT (> 3 months)	0.77	No change	van Agthoven et al. [40]
PD	0.61	0.64	ALCANZA trial
End-stage symptom management care	0.38	No change	Swinburn et al. [41]

Source: CS, Section B.3.4.6. Table 41

*BV* brentuximab vedotin, *ERG* evidence review group, *PD* progressive disease, *PC* physician’s choice, *PFS* progression-free survival, *SCT* stem-cell transplant

Results from the company’s base-case analysis showed that BV dominated the comparison, as it was both cheaper and more effective than PC. The net monetary benefit (NMB) for BV in the company base case was £134,218 at a willingness-to-pay threshold of £30,000 per QALY gained. The most influential parameters in the company’s deterministic sensitivity analyses were the cost of CTCL end-stage care, the utility values of patients 3 months post-alloSCT, the cost of medium Allevyn dressings and the choice of utility value associated with the post-progression health state.

### Critique of the Cost-Effectiveness Evidence and Interpretation

Survival gain in the company base case was due entirely to inclusion of alloSCT in the pathway, as patients treated with BV and PC who did not bridge to alloSCT were assumed to have the same OS. The ERG did not consider it appropriate to include alloSCT in the base-case analysis because of the lack of robust evidence relating to alloSCT effectiveness in the population under consideration, outcomes following alloSCT in patients with advanced-stage CTCL who have received prior treatment with BV and the place of alloSCT in the treatment pathway.

The approach to modelling the post-progression states limited investigation into the impact of varying assumptions about survival. Using means to estimate time spent in the subsequent therapy and end-stage care phases meant that the effect of using different curves to model OS could not be investigated. The approach also meant that some patient populations were modelled to receive very little or no resource-intensive end-stage care if mean time in the post-progression health state was less than the mean time spent receiving subsequent therapy. The ERG considered this to be implausible.

The ERG considered some parameter values and outcomes relating to the post-progression health states to be insufficiently supported by evidence, but it was unable to identify robust alternatives. These parameters and outcomes were as follows: any assumptions that affect the relative time that patients in the BV and PC model arms spent in the post-progression health states, post-progression resource use, and the assumption of equal OS for BV and PC for patients who did not receive alloSCT.

The ERG adjusted the company model to produce a revised base case. Given the limitations of the evidence for the effectiveness of alloSCT, this was not included in the ERG’s revised base case. The ERG recalculated utility values for the pre-progression and post-progression (non-alloSCT) health states using observed utility values from the ALCANZA trial (Table 3) and removed extra AE disutility values. The ERG also removed additional oral chemotherapy costs from the model.

In addition to the revised base case, the ERG also explored the sensitivity of the model to three scenarios: (1) changes to the post-progression pathway, (2) changes to resource-use frequencies and (3) the assumption that, compared with the effectiveness of PC, for patients treated with BV, OS gain was equal to PFS gain (9.5 months). These scenarios were intended to highlight the effect of assumptions in the company base case that had substantial impact on model outcomes but that were not justified by the company or supported by evidence.

### Conclusions of the ERG Report

The ERG considered that results from the ALCANZA trial showed that, compared with PC, treatment with BV resulted in improved ORR4 and PFS but that improvements in OS or HRQoL had not been conclusively demonstrated. Furthermore, peripheral neuropathy was found to be a very common AE for patients treated with BV and, although mostly of low-grade severity, led to treatment discontinuation for approximately one-fifth of all patients treated with BV.

Overall, the ERG considered that patients in the ALCANZA trial with advanced-stage CTCL were similar to patients with advanced-stage MF and pcALCL who would be seen in NHS clinical practice. The ERG highlighted the lack of relative effectiveness evidence for other subtypes of CTCL but acknowledged that obtaining evidence for other subtypes was difficult because CTCL is an orphan disease and given other subtypes constitute less than half of all patients with CTCL.

The ERG’s analyses highlighted the high level of uncertainty around the company base-case cost-effectiveness results. In the revised ERG base case, BV still dominated PC with an NMB of £59,795. When the ERG scenarios were applied to the revised ERG base case individually, the incremental cost-effectiveness ratio (ICERs) per QALY gained varied from £26,331 (changes to resource-use frequencies) to £494,981 (changes to post-progression pathway). In combination, the ERG’s revised base case plus all three scenarios generated an ICER per QALY gained of £125,853. The ERG cautioned that all of the ICERs per QALY gained for the comparison of BV versus PC presented by the company and the ERG may not be reliable given the lack of evidence for many of the key parameters and assumptions.

## National Institute for Health and Care Excellence: Preliminary Recommendation

The AC reviewed the available clinical- and cost-effectiveness data relating to treatment with BV along with testimony from clinical experts and patient representatives at the first AC meeting (December 2018). Following consideration of the evidence presented at the first meeting, the AC did not recommend BV for treating CD30+ CTCL after at least one systemic therapy in adults. The AC noted its preference for varying the rates of alloSCT to reflect uncertainty in clinical practice, varying the number of cycles of BV for patients who receive alloSCT, equal utility values for BV and PC, removing treatment-related disutilities; and removing additional chemotherapy costs. In the ACD, the AC noted its concerns about the clinical- and cost-effectiveness evidence presented at the first meeting, namely, uncertainty over whether BV extends life, either as a bridge to alloSCT or as a treatment without a bridge to alloSCT; that cost-effectiveness estimates were wide ranging; and that none of the cost-effectiveness analyses included all of the AC’s preferred assumptions.

## Independent ERG Report: Response to Consultation

The company submitted further evidence in response to the ACD, including a report and an updated cost-effectiveness model with a revised base case developed using the company’s interpretation of the AC’s preferred assumptions documented in the ACD. Results from the company’s revised base case showed that BV dominated PC and yielded an NMB of £150,415.

The company’s updated cost-effectiveness analysis included modelling post-alloSCT outcomes using an updated cut of the RWD used to estimate post-alloSCT outcomes in the original CS. The ERG’s original concerns about the evidence base for post-alloSCT outcomes also applied to the updated dataset. However, the ERG acknowledged that the AC had concluded that the evidence from the updated RWD was appropriate for decision making.

The ERG noted the lack of justification for some of the outcomes of the company’s revised modelling of PFS and OS following alloSCT, notably that (1) patients whose disease relapsed following alloSCT had substantially worse outcomes than patients whose disease progressed without alloSCT, (2) patients who relapsed following alloSCT did not receive any end-stage care and (3) patients who lived a given number of years after alloSCT without relapsing were assumed to be cured, which equated to a substantial proportion of the alloSCT population. The structure of the model meant that alternative modelling approaches for alloSCT could not be fully investigated; however, the ERG prepared an exploratory scenario to investigate the sensitivity of the ICER per QALY gained to a lower cure rate. This scenario resulted in better life expectancy after relapse than in the company revised base case and allowed patients to receive end-stage care, since mean survival post-progression was longer than the fixed mean time spent receiving subsequent treatment in the model.

The ERG explored three further exploratory scenarios in addition to lowering the cure rate: OS gain for BV in a non-alloSCT population equal to PFS gain (9.5 months), resource-use assumptions as per the ERG’s scenario in its original report, and transplant rate equal to the company’s lower bound estimate (16.7%). When each scenario was applied individually, BV dominated PC, with NMB ranging from £56,584 to 99,672. When applied in combination, the ERG’s four exploratory scenarios yielded an ICER per QALY gained of £58,516.

## National Institute for Health and Care Excellence: Final Guidance

A second AC meeting was held in February 2019 to discuss comments and further evidence received during the consultation period. The FAD was published in March 2019, and final guidance was published in April 2019.

### Clinical Need

The AC heard that CTCL significantly affects HRQoL and that advanced CTCL is associated with poorer prognosis than earlier stages of disease. The AC recognised that there was no existing NICE guidance relating to treating CTCL and heard from clinical experts that treatment options were diverse. The AC also heard from clinicians and patients that there is no uniform response to treatment and that this can have a negative psychological effect on patients. The AC concluded that an unmet need exists for more effective treatment options that both extend the amount of time patients spend in remission from the disease and improve HRQoL.

### Current Practice and Comparators

The AC heard from clinical experts that patients with advanced CTCL are first offered retinoids (bexarotene), IFNα or single-agent chemotherapy (methotrexate) and that BV would be positioned as an alternative to these category A therapies after first-line therapy. The AC concluded that bexarotene, methotrexate and IFNα were the most appropriate comparators. The AC noted that evidence for the efficacy of bexarotene, methotrexate and IFNα was limited and outdated but concluded that it was appropriate to assume, for the purposes of this appraisal, that these treatments were equally efficacious.

### Clinical Effectiveness

The AC noted that the principal clinical-effectiveness evidence had been sourced from the ALCANZA trial (BV vs. PC [bexarotene or methotrexate] for the treatment of CTCL following one previous systemic therapy). The AC did not consider that the absence of direct evidence for the comparison of BV and IFNα was a major limitation since it had previously concluded that bexarotene, methotrexate and IFNα were all equally effective. The AC concluded that the clinical-effectiveness evidence from the ALCANZA trial was relevant to clinical practice in the NHS in England.

The AC recognised that BV improved PFS and had longer clinical responses than bexarotene and methotrexate in the ALCANZA trial and concluded that this would also be the case when compared with IFNα. The AC heard from clinical experts that existing treatments provided short-term responses, so improving response and PFS was clinically meaningful for patients with advanced CTCL. The AC also heard that improved response rates with BV meant that more people could be offered alloSCT.

The AC noted that the company presented a range of data on transplant rates in response to consultation, which were all lower than the transplant rate included in the company base case. The AC concluded that BV could be used as a bridge to alloSCT but that there was uncertainty about the proportion of patients who would receive a transplant in UK clinical practice.

The AC noted that OS data for patients with advanced CTCL in the ALCANZA trial were immature and confounded by treatment crossover, and it acknowledged the company’s justification for assuming no difference in OS between BV and PC for patients who did not receive alloSCT. However, the AC also heard from clinical experts that it might be expected that a substantial PFS gain, such as that seen in the ALCANZA trial, would lead to at least some gain in OS. The AC concluded that there was uncertainty about whether BV would increase OS for patients who did not bridge to alloSCT.

The AC reviewed the supporting data submitted by the company for subtypes of CTCL not included in the ALCANZA trial (SS and LyP). It also considered a statement in the EPAR [31] on the generalisability of efficacy from MF and pcALCL to other subtypes of CTCL. The AC heard from clinical experts that treatments would be similar for most subtypes of CTCL but that they would not use BV for LyP. The AC concluded that clinical-effectiveness data from the ALCANZA trial could be generalised to other subtypes of CTCL, such as SS.

The AC heard from clinical experts that neither the Skindex-29 nor the EQ-5D-3L instruments fully captured the HRQoL impact of advanced CTCL. The AC also acknowledged the views of NHS professionals, submitted during consultation, which emphasised the importance of symptom-burden improvement resulting from treatment with BV. The AC agreed that further research is needed in this area and concluded that, although BV appears to improve HRQoL, the size of the impact is unclear.

### Cost Effectiveness

The AC considered the company model to be structured appropriately for decision making and that it was appropriate to include alloSCT as an option for some patients. The AC concluded that the proportion of patients receiving alloSCT was uncertain but that the company’s lower rate for BV (16.7%) and original rate for PC (7.1%) were acceptable for decision making. The AC acknowledged that data on outcomes following transplant for people with advanced CTCL were limited but concluded that the company’s approach to modelling outcomes after alloSCT was appropriate for decision making.

The AC noted that the modelling of OS for patients who did not receive alloSCT was uncertain. The AC understood that cost-effectiveness estimates in scenarios that included an OS gain for patients treated with BV were much higher than in the company base case but noted that there was no evidence to show what OS gain was most likely in clinical practice. However, given the effect on the post-progression pathway of assuming an OS gain, the AC concluded that it should consider an OS gain equal to PFS gain (9.5 months) in its decision making for patients who did not receive alloSCT.

The AC considered that the ERG’s preferred pre-progression utility value for both BV and PC (0.69) was more appropriate than the values used in the company base case. The AC also considered that the ERG’s resource-use scenario should be used in the cost-effectiveness analysis, as it better reflected end-stage care for most patients with advanced CTCL. The AC also acknowledged that the cost-effectiveness results were not sensitive to the length of time patients received BV before bridging to transplant.

Applying the AC’s preferred assumptions (i.e. transplant rate of 16.7%, OS gain of 9.5 months with BV and using the ERG's resource-use scenario) generated an ICER for BV versus PC of £29,613 per QALY gained. Thus, the AC concluded that the most plausible ICER for BV compared with PC was less than £30,000 per QALY gained, which is within the range normally considered an acceptable use of NHS resources.

### End-of-Life Criteria

The AC noted that the company had not provided a case for BV to be considered a life-extending treatment at the end of life, that clinical experts did not consider BV to fulfil criteria for end of life and that data presented by the ERG did not support BV as an end-of-life treatment. The AC accepted that BV did not meet end-of-life criteria.

### Final Guidance

The AC recommended BV as an option for treating CD30+ CTCL after at least one systemic therapy in adults with MF stage IIB or higher, pcALCL or SS and if the company provides BV according to the commercial arrangement (i.e. simple discount PAS).

## Conclusion

The principal challenge in this appraisal was the lack of evidence for many key parameters, particularly following progression or involving alloSCT. Cost-effectiveness estimates were very sensitive to relatively small changes in some parameters, such as relative time spent by patients receiving end-stage care, for which neither the company nor the ERG could present robust estimates for UK clinical practice. This uncertainty was compounded by the structure of the model for post-progression health states, which traded the flexible but complex structure of a partitioned survival model for the simplified but more rigid pay-off design.
